# *Mansonella ozzardi* mitogenome and pseudogene characterisation provides new perspectives on filarial parasite systematics and CO-1 barcoding

**DOI:** 10.1038/s41598-018-24382-3

**Published:** 2018-04-18

**Authors:** James Lee Crainey, Michel Abanto Marín, Túllio Romão Ribeiro da Silva, Jansen Fernandes de Medeiros, Felipe Arley Costa Pessoa, Yago Vinícius Santos, Ana Carolina Paulo Vicente, Sérgio Luiz Bessa Luz

**Affiliations:** 1 Instituto Leônidas e Maria Deane/ILMD/FIOCRUZ, Laboratório de Ecologia de Doenças Transmissíveis na Amazônia, Manaus, Amazonas, Brazil; 2 Fundação Oswaldo Cruz, Instituto Oswaldo Cruz, Laboratório de Genética Molecular de Microrganismos/IOC/FIOCRUZ, Rio de Janeiro, Rio de Janeiro, Brazil; 3 Fundação Oswaldo Cruz, FIOCRUZ Rondônia, Laboratório de Entomologia, Porto Velho, Rondônia, Brazil; 4 Programa de Pós-Graduação em Biologia da Interação Patógeno Hospedeiro (PPGBIO-Interação), Manaus, Amazonas, Brazil; 5 Scientific and Technological Bioresource Nucleus (BIOREN) University of La Frontera, Temuco, Chile

## Abstract

Despite the broad distribution of *M. ozzardi* in Latin America and the Caribbean, there is still very little DNA sequence data available to study this neglected parasite’s epidemiology. Mitochondrial DNA (mtDNA) sequences, especially the cytochrome oxidase (CO1) gene’s barcoding region, have been targeted successfully for filarial diagnostics and for epidemiological, ecological and evolutionary studies. MtDNA-based studies can, however, be compromised by unrecognised mitochondrial pseudogenes, such as Numts. Here, we have used shot-gun Illumina-HiSeq sequencing to recover the first complete *Mansonella* genus mitogenome and to identify several mitochondrial-origin pseudogenes. Mitogenome phylogenetic analysis placed *M. ozzardi* in the Onchocercidae “ONC5” clade and suggested that *Mansonella* parasites are more closely related to *Wuchereria* and *Brugia* genera parasites than they are to *Loa* genus parasites. DNA sequence alignments, BLAST searches and conceptual translations have been used to compliment phylogenetic analysis showing that *M. ozzardi* from the Amazon and Caribbean regions are near-identical and that previously reported Peruvian *M. ozzardi* CO1 reference sequences are probably of pseudogene origin. In addition to adding a much-needed resource to the *Mansonella* genus’s molecular tool-kit and providing evidence that some *M. ozzardi* CO1 sequence deposits are pseudogenes, our results suggest that all Neotropical *M. ozzardi* parasites are closely related.

## Introduction

*Mansonella ozzardi* is one of three species of filarial parasite from its genus known to commonly use humans as a definitive host and to occur widely throughout Latin America and the Caribbean^1–3^. In the Brazilian Amazon, *M. ozzardi* is known to be transmitted by a wide range of insects including blackflies from the genus *Simulium*. In the Caribbean and elsewhere, however, the only known vectors are from the family Ceratopogonidae^2,3^. For this reason, it has been hypothesised that the *M. ozzardi* from the Caribbean region are distinct from the parasites of continental South America^4–6^.

Mitochondrial genes have been used extensively for diagnostics, systematics and population studies^7–11^. The haploid nature and fast rate of evolution of the CO1 gene has made it a particularly popular target for these studies and for these reasons also the target of an international DNA barcoding project that has been widely used for molecular taxonomy^12,13^. Mitochondrial DNA can, however, sometimes integrate into nuclear genomes and become pseudogenes, often referred to as Numts^14–16^. Unidentified mtDNA pseudogenes, like Numts, pose a threat to reliable molecular diagnostics, systematics and population analyses that include them^17–20^.

In this study, we report the complete mitochondrial genome of *M. ozzardi* parasites isolated from microfilariae obtained from an infected resident of Tefé, an Amazonas State town situated on the banks of the Rio Solimões, Brazil. In addition to providing a potentially useful new resource to the human filarial parasite community, this sequence has helped us to identify 14 novel mtDNA pseudogene sequences (MG913165- MG913178) and to incriminate previously published GenBank CO1 sequence deposits as potential pseudogenes (JF412329–JF412347). In the results we present here we also show that Caribbean and South American *M. ozzardi* are very closely related and most likely are not separate species as has been previously proposed^4–6^.

## Methods

### *Mansonella ozzardi* sample collection and identification

In previous work blood samples were taken from volunteers living in and around the Brazilian Amazon village of Tefé (3° 22′ 00″ S; 64° 42′ 00″ W), following a protocol (1504/10) approved by the Research Ethics Committee of the Fundação de Medicina Tropical Doutor Heitor Vieira Dourado, Manaus^21^. *Mansonella ozzardi* positive blood samples were identified as containing only *M. ozzardi* parasites by light microscopy (using methods described in Post *et al*.^22^) and by PCR amplification and sequencing of four taxonomic identifier sequences: a partial mitochondrial 12 S sequence, a partial CO1 gene sequence, a partial nuclear ribosomal 5 S sequence and a sequence spanning the complete ribosomal ITS-1 sequence. These PCR reactions followed previously published protocols and were all performed on microfilarial DNA extracts obtained from blood samples using QIAGEN blood and tissue kits^8,21,23^.

### Shotgun Reversible Dye Terminator DNA sequencing of the *M. ozzardi* mitogenome

A single microfilarial DNA extract (containing a pool of microfilariae) obtained from a single blood collection taken from a single Tefé resident that tested positive for *M. ozzardi* (and no other filarial parasite) in both light-microscopy and PCR testing, was selected for *M. ozzardi* mitogenome characterisation. Shot-gun DNA sequencing of this DNA extract was performed on an Illumina HiSeq 2500 system (Oswaldo Cruz Foundation, high-throughput sequencing platform) using 2 × 100 nucleotide paired-end reads generated with Nextera Truseq libraries. Reads corresponding to human-host DNA were filtered out by mapping them against a human reference genome (accession number: GCA_000001405.19). The *M. ozzardi* mitogenome was assembled with the A5-miseq pipeline^24^. Mapping and short read post-processing was performed using Bowtie2 software^25^ and Samtools utilities^26^, respectively. The prediction of protein-coding genes, rRNAs and tRNAs was done using MITOS^27^ and Arwen software^28^ and validated manually by comparing homologous regions with a *Loa loa* mitochondria reference in Artemis^29^. A mitogenome map was generated using the BRIG software package^30^.

### Mitogenome sequence assembly verification

A provisional mitogenome nucleotide sequence assembly was aligned to a set of 21 previously published nematode mitogenome sequences using CLUSTAL X^31^ (See supplementary information, Fig. 1). This identified an anomalous region in our provisional mitogenome assembly which was PCR amplified using primers designed from one conserved part of the NADH dehydrogenase subunit 2 gene (NADHDS2: TGAATGTGGTTTTGTTGTTCCT) and one conserved part of the NADH dehydrogenase subunit 5 gene (NADHDS5: GGGCTGCTATAGCCTTTGGT). Amplification was performed with a PCR reaction mix containing: 0.5 µl of each primer at a concentration of 100 mM, 5 µl of *M. ozzardi* DNA extract and 16.5 µl of PCR master mix in a 50 µl reaction mix, topped-up with 27.5 µl ultrapure sterilised water. The chemical composition of the PCR master mix was: 10 µl of 5 × Promega GoTaq PCR buffer; 4 µl of MgCl2 at a concentration of 25 mM and 1 µl of a DNTP mix, containing each DNTP at a concentration of 10 mM, and 1.5 µl of promega GoTaq at a concentration of 5Uµl-1. PCR amplification used the following cycling conditions: 95 °C for 2 minutes followed by 5 cycles of: 95 °C for 1 minute then 58–68 °C for 1 minute then 72 °C for 1 minute and 30 seconds; followed by 40 cycles of: 95 °C for 1 minute then 58 °C for 1 minute then 72 °C for 1 minute and 30 seconds. The protocol was completed with a final extension step of 72 °C for 10 minutes. Amplified PCR products were Sanger sequenced in both directions using the forward and reverse NADHDS2 NADHDS5 primers used to amplify them as well as a set of internal primers: TGTGGTTTTGTTGTTCCTAGTTT and AAAACCCAATCACAGACAATGAA. Prior to sequencing, the amplicons were cleaned-up using a Promega wizard PCR kit and protocol. Sequencing was done with an Applied Biosystems BigDye Terminator kit and ABI PRISM® 3100 Genetic Analyzer.

**Figure 1:**
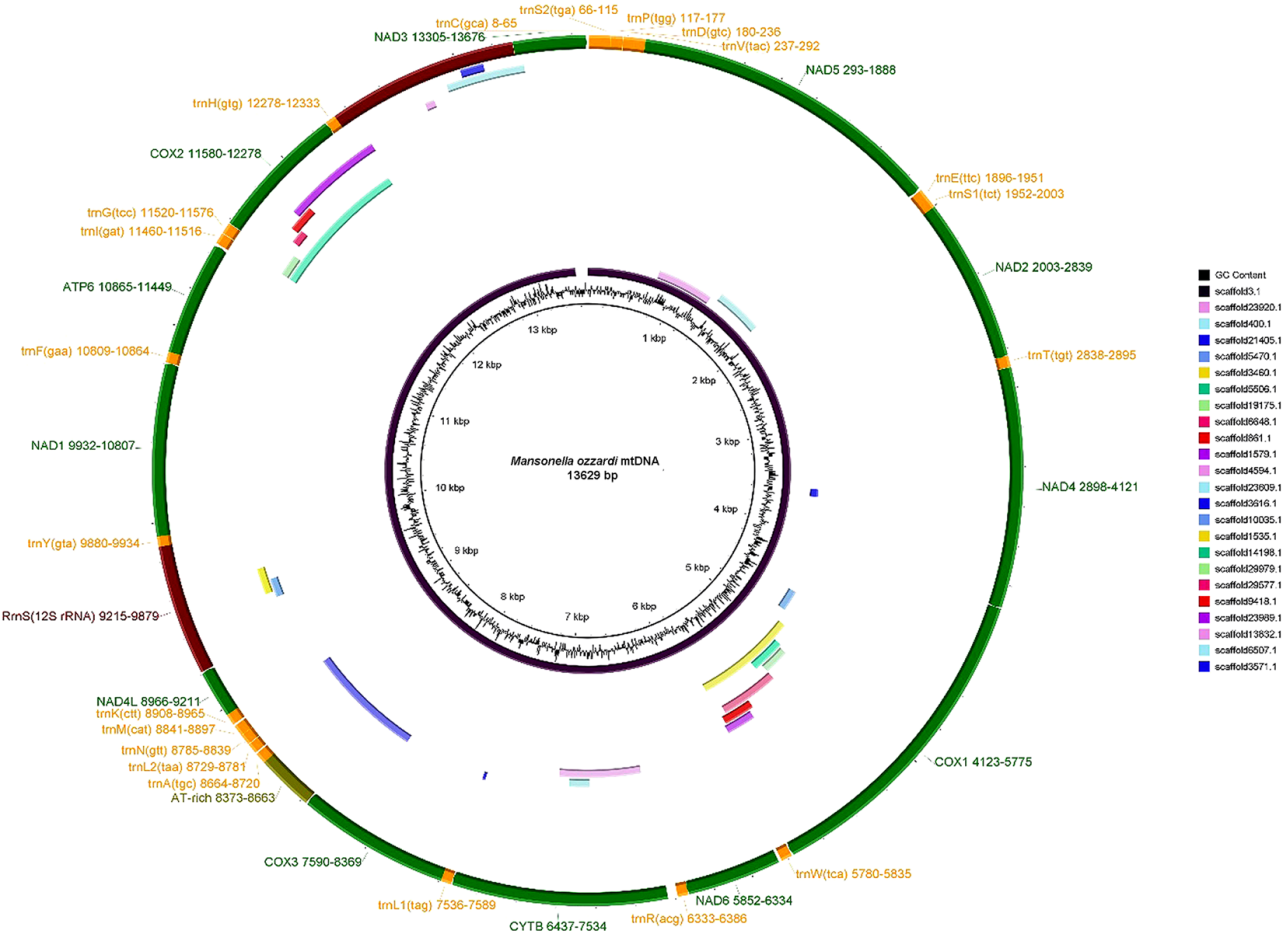
The genomic architecture of the *M. ozzardi* mitogenome shows a graphical representation of the *M. ozzardi* genome. The mitogenome’s annotated with gene content, gene order and orientation. Inside the circular genome proposed mitogenome Numt sequences (described in detail in Table 1) are mapped to the genome.

### Phylogenetic analysis

DNA and conceptually translated amino acid sequences obtained for this study were aligned with sequences downloaded from GenBank using the program Clustal X 2.0^31^. Sequence alignment editing was done in GenDoc^32^. Phylogenetic analysis was performed using software from the PHYLIP package (version 3.67)^33^. Because analysis of a similar set of filarial parasite mitogenomes found evidence that variation in the third codon base position of mitochondrial protein coding regions had reached saturation (see Yilmaz *et al*.^34^), our whole mitogenome tree construction was done using an alignment of conceptually translated amino acid sequence concatemers and the Maximum likelihood tree-building program “proml” of Felsenstein^33^. The mitogenome alignment used for this tree was prepared with 22 mitogenome sequences and all 12 mitochondrial protein codon gene sequences (see supplementary information, Fig. 2). In order to make our phylogenetic analysis easily comparable with previous analysis of Yilmaz *et al*.^34^ we used mitogenome sequences from all of the species that they used in their analysis and thus have included mitogenome sequences from two nematode species that are not from the filarial worm taxa Onchocercidae. Yilmaz *et al*.^34^ used *Ascris lumbricoides* and *Dracunculus medinensis* mitogenome sequences as outgroups in the construction of their phylogenetic tree and so we have also included these non-Onchocercidae nematode-origin sequences in our analysis. Maximum likelihood trees from partial mitochondrial CO1 and 12 S sequences were prepared using nucleotide-level alignments and the PHYLIP program “dnaml”, as was the nuclear 5 S ribosomal tree^33^. The robustness of all four tree topologies was tested with 1,000 bootstrap pseudo replicate alignments that were generated with the PHYLIP program “seqboot”^33^.

## Results

### The complete mitogenome of *M. ozzardi* has an architecture typical of a filarial parasite mitogenome

Our initial assembly of the *M. ozzardi* mitogenome used standard illumina Hiseq sequence reads and sequence assembly software. The resultant provisional *M. ozzardi* mitogenome was of 13,681 nucleotides in length and was seen to share remarkable genomic architectural similarities with previously reported filarial parasite mitogenomes. The *M. ozzardi* mitogenome encodes exactly the same set of genes, arranged in the same order and orientation, as all hitherto published Onchocercidae mitogenomes (see Fig. 1). This astonishing level of synteny, allowed for us to easily align the raw nucleotide sequence of our provisional *M. ozzardi* mitogenome to the 21 previously determined filarial parasite mitogenomes. This suggested that our provisional *M. ozzardi* genome contains a 52 nucleotide sequence insert that is not present in any of the other previously published filarial parasite mitogenomes. The apparent insert was observed to occur between the two genes that encode the NADHDS2 and NADHDS5 proteins (see supplementary figure 3). As the Illumina Hiseq sequence run had also detected the occurrence of pseudogenes sequences covering this region (see Fig. 1 and Table 1), which we recognised could potentially corrupt our mitogenome assembly, we decided to Sanger sequence across this region to determine whether this 52 nucleotide region represented an artefact of mitogenome assembly. PCR primers designed from conserved regions of the NADH dehydrogenase subunits 2 and 5 were used to amplify and sequence this region and determined that this 52 nucleotide sequence was indeed an artefact of sequence assembly. Figure 1 shows the mitogenome organisation of *M. ozzardi*, which has the same architecture as all hitherto reported filarial parasite genomes^34^. The complete and verified mitogenome sequence of *M. ozzardi* is 13,629 base pairs and has been deposited at GenBank and assigned the accession number KX822021.

**Table 1:** *Mansonella ozzardi* mtDNA sequences

Accession number	Coordinates and length (in brackets)	Description
MG913165	9,611–10, 481(897 nucleotides)	This mtDNA pseudogene sequence shares 84% identity (751/897) with the *M. ozzardi* mitogenome. The shared identity spans the *M. ozzardi* t-RNA gly gene, all of the COX-2 gene, all of the t-RNA His gene, and the first 46 nucleotides of the 16 S rDNA genes. It has been categorised as a pseudogene because of the low level of identity it shares with the reference genome and because it contains 4 indels in its COX-2-like sequence region. Two of the indels are single nucleotide insertions and one is a single nucleotide deletion. The fourth is a 19-nucleotide insertion. This contig´s COX-2-like sequence “codes” for 6 stop codons.
MG913166	6,225–7, 006(790 nucleotides)	This mtDNA pseudogene sequence shares 83% identity (652/788) with the *M. ozzardi* mitogenome. The identity spans from the middle of the COX-3 gene to the end of the tRNA-Met gene. It also spans the whole tRNA-Ala, tRNA-Leu and tRNA-Asn genes. Although there are no indels or strop codons “encoded” in the 240 nucleotides in the contigs COX-3 gene like sequence, this contig has been categorised as pseudogene because it shares such a low level of identity with the mitogenome and because it contains 10 indels. The indels occur in its tRNA gene coding regions: four single nucleotide insertions, one single nucleotide deletion, two 2-nucleotide deletions, two 2-nucleotide insertions and a single 7-nucleotide deletion.
MG913167	12,488–13, 078(591 nucleotides)	This mtDNA pseudogene sequence shares 92% identity (545/591) with an internal portion of the NADH dehydrogenase subunit 5. It contains only one indel: a single nucleotide insertion. The single nucleotide insertion causes a frameshift mutation but no stop codons are “encoded” before.
MG913168	4,566–5,148(585 nucleotides)	This mtDNA pseudogene sequence shares 83% identity (482/578) with the *M. ozzardi* mitogenome. It shares identity with a 578 nucleotide portion of the cytochrome b gene. It contains two single nucleotide indels: one insertion, one deletion, but does not appear to encode stop codons.
MG913169	(584 nucleotides)3,292–3, 873	This mtDNA pseudogene sequence shares 86% (501/583) with the *M. ozzardi* cytochrome c oxidase subunit I (CO1) sequence. It contains four indels: two single nucleotide deletions, a single 2-nucleotide insert and a single 8-nucleotide deletion. It also appears to encode two stop codons.
MG913170	(576 nts)9,937–10, 510	This mtDNA pseudogene sequence shares 85% (487/576) with *M. ozzardi* mitogenome. Four single nucleotide indels: two insertion, two deletions. The first 446 nucleotides match last 446 of the cytochrome c oxidase subunit II gene (COII). The COII genes matching region contains one stop codon and one single nucleotide deletion. The contig also spans the tRNA-His gene and the first 76 nucleotides of the 16 S gene. The contig share 86% identity (489/566) with contig 14198.
MG913171	(471 nts)13,150–13, 617	This mtDNA pseudogene sequence shares 86% (403/471) with the coding region of NADH dehydrogenase subunit 5. It also contains seven indels: three single nucleotide deletions, three 3-nucleotide deletions, and a single 2-nucleotide deletion.
MG913172	(464 nts)3,356–3, 819	This mtDNA pseudogene sequence shares 80% (371/464) with a portion of the cytochrome c oxidase subunit I (CO1). It contains four indels: three single nucleotide deletions, one 8-nucleotide deletion. It also encodes stop codon.
MG913173	(432 nts)3,292–3, 873	This mtDNA pseudogene sequence shares 86% (501/583) portion of the cytochrome c oxidase subunit I. It contains three indels: one single nucleotide insertion, one single nucleotide deletion and one seven nucleotide deletion.
MG913174	(424 nts)11020–11444	This mtDNA pseudogene sequence shares 89% (382/427) identity with the *M. ozzardi* mitogenome sequence. It matches the last 378 16 S ribosomal RNA and contains five indels: two single nucleotide deletions, one 3-nucleotide deletion, one 6-nucleotide deletion and a single 2-nucleotide insertion.
MG913175	3,123–3, 411(292 nts)	This mtDNA pseudogene sequence shares 89% (261/292) identity with the *M. ozzardi* mitogenome cytochrome c oxidase subunit I (CO1) gene. It contains a three nucleotide insertion.
MG913176	3,618–3, 857(240 nts)	This mtDNA pseudogene sequence shares 91% (219/240) identity with the *M. ozzardi* mitogenome cytochrome c oxidase subunit I. It contains no indels or stop codons.
MG913177	(220 nts)3,655–3, 872	This mtDNA pseudogene sequence shares 79% (173/220) identity with the *M. ozzardi* mitogenome cytochrome c oxidase subunit I (CO1) gene. It encodes three indels:one single nucleotide insertion, deletions and two nucleotide deletions: one four nucleotides in length and one seven in length.
MG913178	(210 nts)3,146–3, 355	This mtDNA pseudogene sequence shares 84% (176/210) identity with the *M. ozzardi* mitogenome cytochrome c oxidase subunit I (CO1) gene. It encodes two indels: two single nucleotide deletions. It also ‘encodes’ and two stop codons.

An inventory of 14 mtDNA pseudogene sequences over the length of 200 nucleotides that have been identified from a *M. ozzardi* illumina HiSeq contig library. As well as providing GenBank sequence accession numbers and the length of each of the mtDNA pseudogene sequences, the table also provides the basis on which these sequences were categorised as pseudogene sequences.

### *Mansonella ozzardi* belongs to the Onchocercidae “ONC5” clade and maybe more closely related to the lymphatic filariasis causing parasites than previously thought

**Figure 2:**
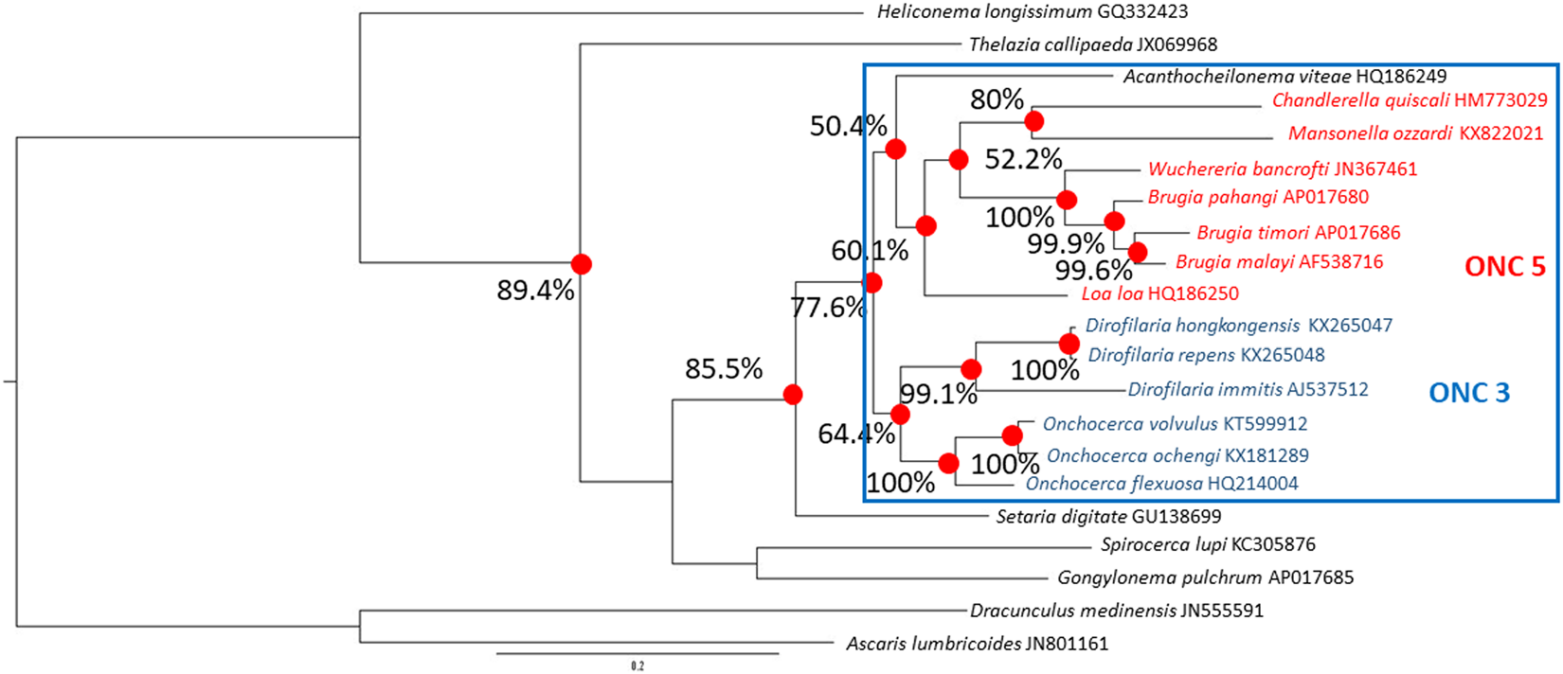
A phylogenetic tree constructed from filarial parasite mitogenome sequences. A representative maximum likelihood tree constructed from a 3378 amino acid residue alignment prepared by concatenating all 12 mitochondrial protein sequences from the *M. ozzardi* mitogenome and aligning to their orthologous protein sequences from 21 other whole mitogenome protein sequences from allied parasitic nematodes, (this alignment is available in the supplementary information, Fig. 3). Bootstrap-supported nodes referred to in the text are indicating with percentage values that were determined from 1000 pseudoreplicates. Branch labels provide source species names and GenBank accession numbers from which whole mitogenome sequence can be retrieved.

Figure 2 shows a phylogenetic tree constructed from the protein coding region of 21 parasitic nematode worms. This tree was constructed using a similar approach to Yilmaz *et al*.^34^ and is very similar to theirs. Our analysis, like the analysis of Yilmaz *et al*.^34^, supports the seven-gene multi locus sequence typing (MLST) analysis of Lefoulon *et al*.^35^ and therefore strongly supports the monophyly of Onchocercidae and the existence of at least two of the phylogenetic Onchocercidae clades (ONC4 and ONC5) within it. Consistent also with the MLST analysis of Lefoulon *et al*.^35^, our mitogenome analysis has placed *M. ozzardi* in the “ONC5” clade. As in both the MLST analysis of Lefoulon and the mitogenome analysis of Yilmaz *et al*.^34^, in our analysis all four lymphatic filariasis causing parasites (*W. bancrofti*, *B malayi*, *B. timori, B. pahangi*) have clustered together in a highly boot-strap supported monophyletic group. In the mitogenome analysis of Yilmaz *et al*.^34^ the relationship between this grouping and the other ONC5 clade filarial parasites was not resolved. In our analysis the *L. loa* parasite forms an out-group to a weekly bootstrap-supported monophyletic group containing two highly bootstrap-supported sister clades. One of these sister clades contains the four parasites that cause lymphatic filariasis and the other contains *M. ozzardi* and the chicken filarial parasite *Chandlerella quiscali*. Our analysis therefore suggests that *M. ozzardi* is more closely related to the four parasite species that cause lymphatic filariasis than it is to the *L. Loa* parasite (which causes Loiasis). It should, however, be noted that while the clustering supporting this observation has bootstrap support (52.2%), such weak support is not always considered significant and is certainly far from the >90% bootstrap support that would typically be considered as robust support.

### The identification of mitochondrial pseudogenes from a *M. ozzardi* DNA shot-gun sequence contig library

Using the provisionally assembled *M. ozzardi* mitogenome as a query in a BLASTn search of a *M. ozzardi* DNA shot-gun sequence contig library we identified 24 contigs that we have classified as pseudogenes. Fourteen of these contigs range between ~200 and 800 nucleotides in length and not only share significant nucleotide identity with our *M. ozzardi* mitogenome sequence, but also display the characteristic hallmarks of Numt pseudogenes (i.e they encode frame-shift mutations or stop codons or contain indels— see Table 1). We have deposited these sequences as putative mtDNA pseudogenes *M. ozzardi* sequences at GenBank, where can be retrieved using their assigned accession numbers: MG913165-MG913178. Table 1 provides an inventory of the mtDNA pseudogenes sequences and their accession numbers and also provides details on what basis each sequence was categorised as a pseudogene sequence. In Fig. 1 these contigs are displayed mapped to the circular mitogenome of *M. ozzardi*. As can be seen in Fig. 1 (and also Table 1) the pseudogene sequences identified in this study cover a broad range of *M. ozzardi* mitogenome. Notably, half of the mtDNA pseudogene contigs listed in Table 1 share identify with the CO1 sequence cytochrome oxidase I (CO1) gene, a gene which is often used as a barcode for species identifications (see below). Notably too, two putative pseudogene sequences with sequence identity spanning the region between NADHDS2 to NADHDS5 (the region which contained a putative 52 nucleotide insertion in our provisional mitogenome assembly) were also identified. We did not identify mtDNA pseudogenes sharing identity with the 12 S mitochondrial gene.

### The characterisation of CO1 pseudogenes from GenBank sequence deposits: Peruvian CO1 sequences are genetically distinct from Haitian and Brazilian CO1 sequences

**Figure 3:**
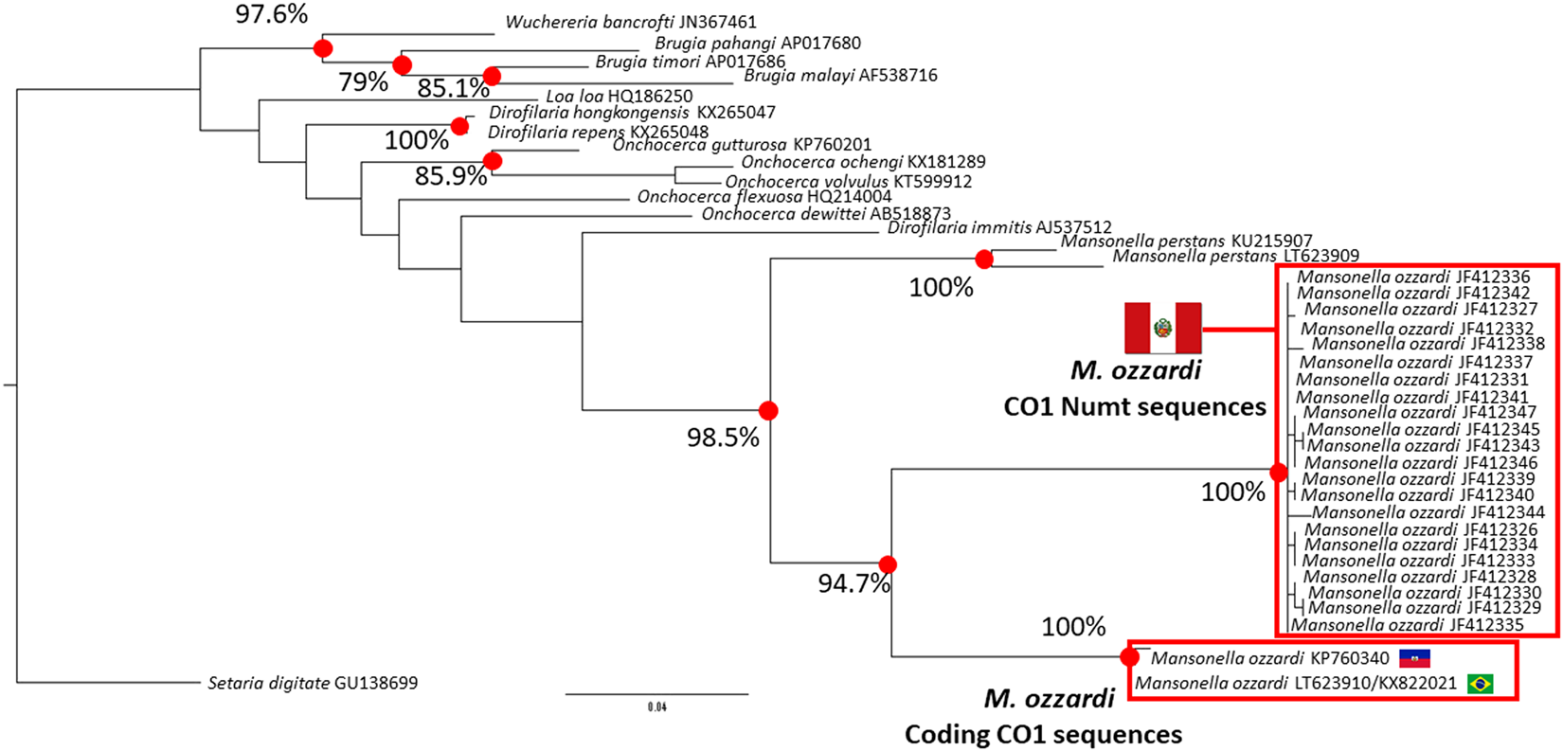
A phylogenetic tree constructed from filarial parasite CO1 sequences. A representative maximum likelihood tree constructed from an alignment of partial mitochondrial and Numt CO1sequence spanning 601 nucleotide positions (available in the supplementary information, Fig. 4). Bootstrap-supported nodes, referred to in the text, are indicated with percentage values that were determined from 1000 pseudoreplicates. Branch labels provide source species names and GenBank accession numbers, which can be used to retrieve source sequences. National flags are used to indicate the geographical origin of where *M. ozzardi* CO1 sequences derive.

Figure 3 show a filarial parasite phylogenetic tree constructed with a partial mitochondrial DNA sequence from the cytochrome oxidase gene (CO1). This tree has been constructed with an alignment made using a partial *M. ozzardi* CO1 sequence obtained from its mitogenome (KX822021) and various reference sequences retrieved from GenBank sequence deposits. As can be seen in Fig. 3, the *M. ozzardi* CO1 sequence obtained for this study (KX822021) clusters in a 100% bootstrap supported monophyletic group with a *M. ozzardi* CO1 reference sequence (KP760340) obtained from Haitian *M. ozzardi*. This Brazilian-Haitian group forms a sister clade to a 100% bootstrap supported cluster of 21 CO1 sequences obtained from Peruvian *M. ozzardi* samples and collectively these two clusters form a bootstrap-supported monophyletic group that is a sister clade to a *M. perstans* specific cluster. The topology of the tree thus suggest that the *M. ozzardi* CO1 sequences from Haiti and Brazil share common ancestry before either does with the Peruvian CO1 sequences and also that all the *M. ozzardi* CO1 sequence share common ancestry with one another before any do with *M. perstans* CO1 sequences.

**Figure 4:**
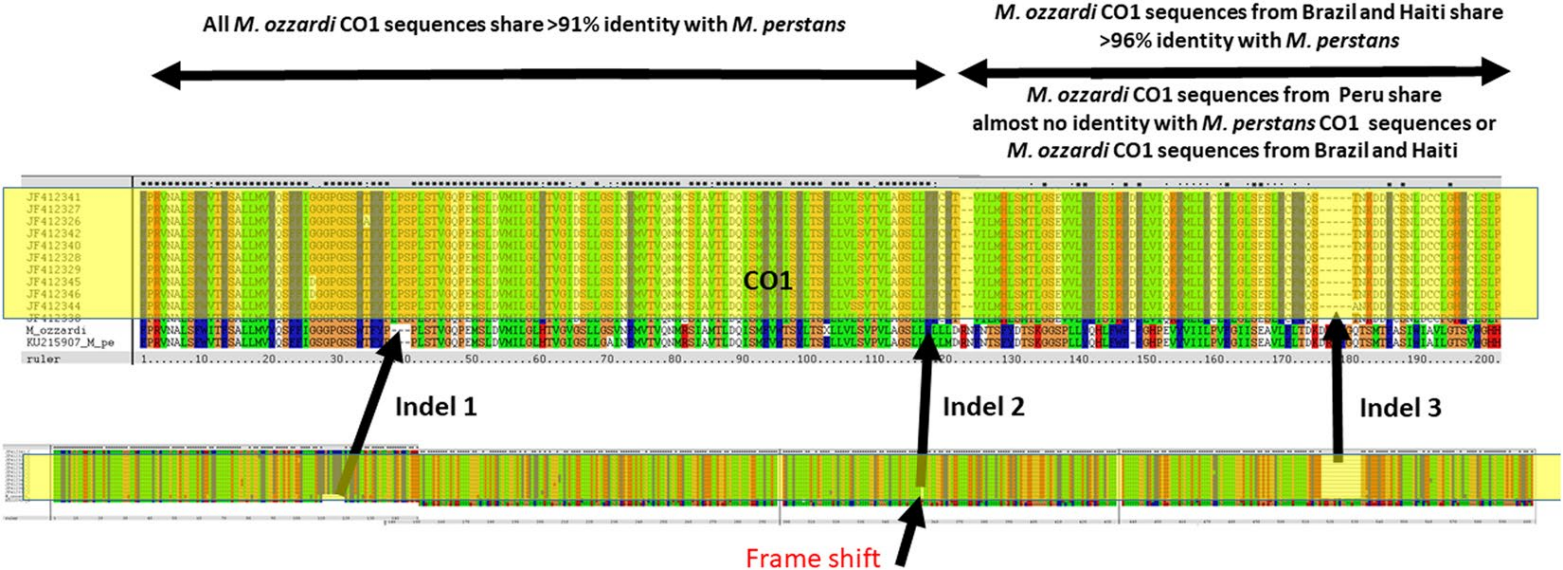
Conceptual translations of *M. ozzardi* CO1 sequence deposits Shows an amino acid level alignment of conceptually translated *M. ozzardi* CO1 sequences. Variant *M. ozzardi* CO1 sequences from Peru are labelled with their source nucleotide sequence accession numbers and are shown aligned to a conceptually translated *M. perstans* CO1 reference sequence and the *M. ozzardi* CO1 sequence generated for this study. The source nucleotide sequences used to create the amino acid level alignment are shown below it. Three indels discussed in the text of the paper are highlight, as is a break in identity caused by a frame-shift mutation. Yellow highlighting has been used to make the grouping of Peruvian CO1 sequences more obvious.

The genetic distance between the Peruvian and the Brazilian and Haitian CO1 sequence clusters is, however, comparable to the genetic distance between species and even genera. Across the 601 nucleotide positions used in the alignment to construct the tree shown in Fig. 2, the *M. ozzardi* from Haiti and Brazil share > 99% identity, but neither the Haitian or the Brazilian sequences share >84% identity with their closest CO1 relatives from Peru. The genetic distance between the Brazilian-Haitian and the Peruvian *M. ozzardi* CO1 cluster is thus greater than the distance between the *Wuchereria bancrofti* CO1 sequence and its closest relative (AP017686) from the *Brugia* genus, which share >92% identity with one another. In Supplementary Fig. 3, part of this genetic distance between the *M. ozzardi* CO1 clusters can been seen to be attributable to what we contend here are three indels occurring in the CO1 sequences of Peruvian *M. ozzardi* CO1 sequences. None of these proposed indels occur in the alignment of any of the other filarial parasite CO1 sequences. The first of these is a nine nucleotide insertion, which does not produce a frame shift in the corresponding amino acid sequence. The second is a single nucleotide deletion and the third is a 16 nucleotide deletion. Using conceptual translation of each of the variant *M. ozzardi* CO1 sequences (ie the Brazilian, Haitian and Peruvian CO1 sequences) and aligning them to a reference *M. perstans* CO1 amino acid sequence (ANE06625) the single nucleotide deletion can be seen to correspond with a marked fall-off in amino-acid identity. Conceptual translation of the 354 nucleotides that immediately precede this gap, results in a 114 amino acid sequence that share at least 91% identity with the *M. perstans* CO1 amino acid (ANE06625) reference sequence (See Fig. 4). Although conceptual translation of the 230 nucleotides in the Peruvian CO1 sequences immediately following this deletion does not result in the introduction of a stop codon the resultant amino acid sequence shares almost no identity with the *M. perstans* CO1 reference sequence. In contrast to this, conceptual translation of the corresponding 246 nucleotides of the Brazilian and the Haitian CO1 *M. ozzardi* sequences results in amino acid sequences that share at least 96% identity with the *M. perstans* CO1 sequences. The CO1 sequences of Peruvian *M. ozzardi* can thus be regarded as containing a cryptic frame-shift mutation and displaying many of the hallmarks of non-coding mtDNA pseudogenes Numt sequences, which have integrated into the genome of *M. ozzardi* after its divergence from *M. perstans*.

### The characterisation of CO1 pseudogenes from GenBank sequence deposits: 5 S and 12 S reference sequences suggest Peruvian *M. ozzardi* are almost identical to Brazilian, Bolivian and Haitian parasites

**Figure 5:**
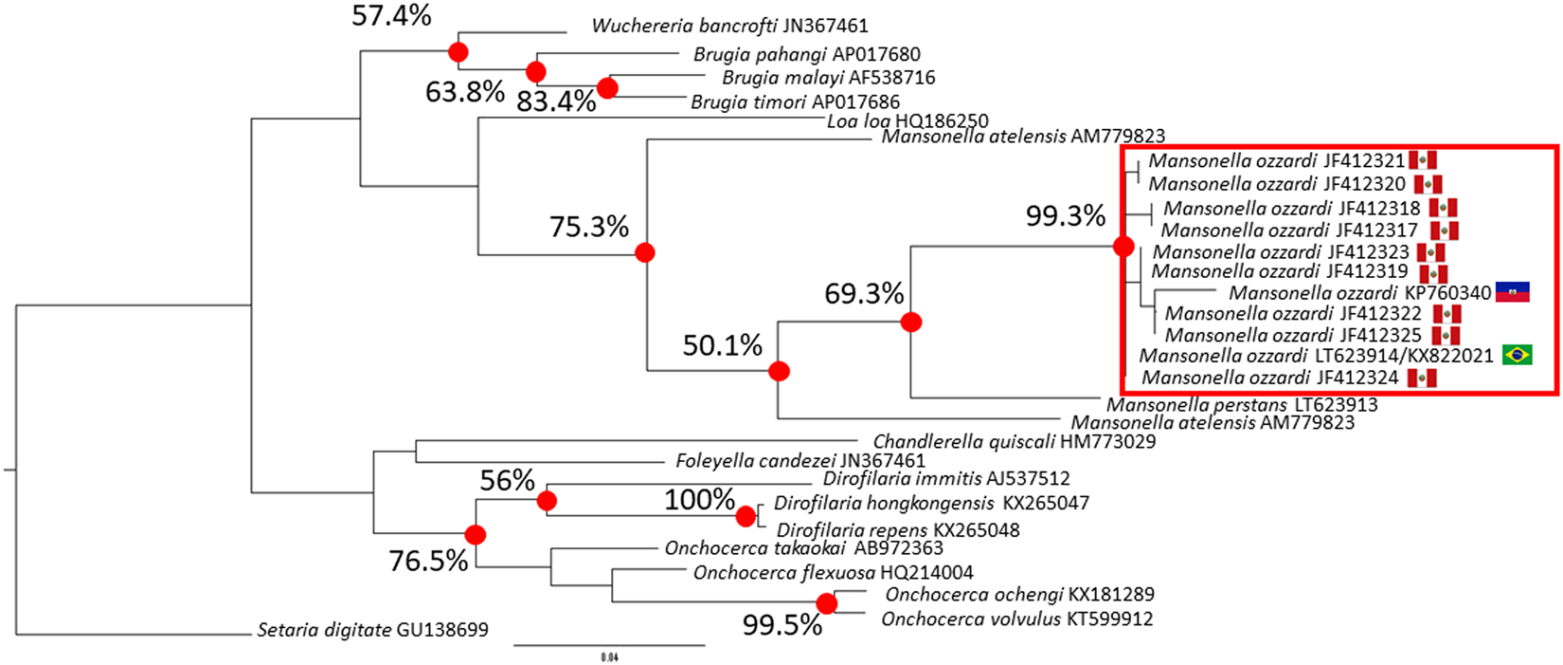
A phylogenetic tree constructed from filarial parasite 12 S sequences. A representative maximum likelihood tree constructed from an alignment of partial mitochondrial 12 S sequences spanning 380 nucleotide positions (available in the supplementary information, Fig. 4). Bootstrap-supported nodes, referred to in the text, are indicated with percentage values that were determined from 1000 pseudoreplicates. Branch labels provide source species names and GenBank accession numbers, which can be used to retrieve source sequences. National flags are used to indicate the geographical origin of where *M. ozzardi* 12 S sequences derive.

**Figure 6:**
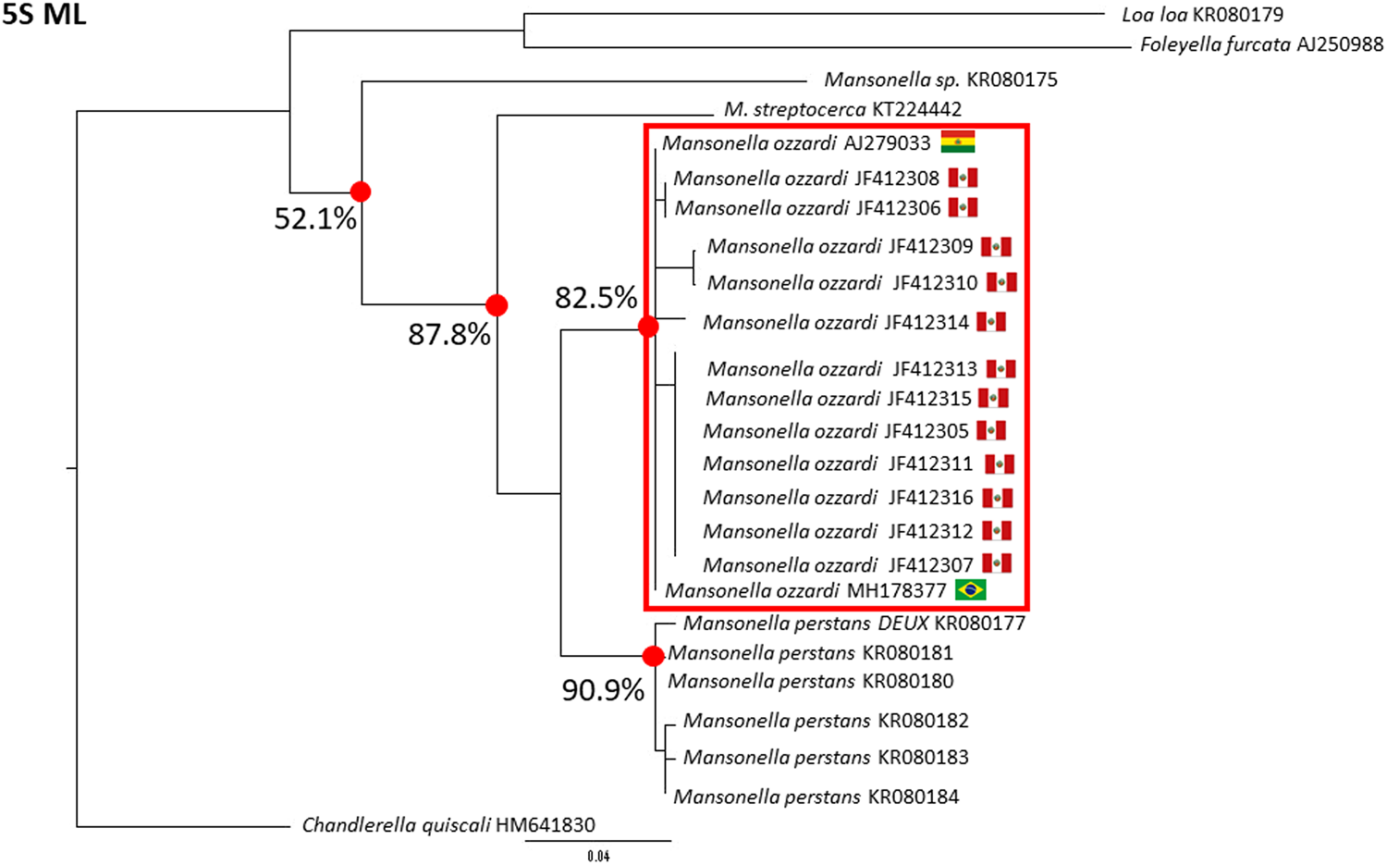
A phylogenetic tree constructed from filarial parasite 5 S sequences. A representative maximum likelihood tree constructed from an alignment of partial ribosomal 5 S sequences spanning 380 nucleotide positions (available in the supplementary information, Fig. 5). Bootstrap-supported nodes, referred to in the text, are indicated with percentage values that were determined from 1000 pseudoreplicates. Branch labels provide source species names and GenBank accession numbers, which can be used to retrieve source sequences. National flags are used to indicate the geographical origin of where *M. ozzardi* 5 S sequences derive.

Although the Peruvian *M. ozzardi* CO1 sequences show many of the hallmarks of mtDNA pseudogene Numt origin, their discrete grouping in Fig. 3 could also be explained by them deriving from an a-typical strain of *M. ozzardi*, a novel species or *M. ozzardi* supporting multiple mitochondrial haplotypes. A-typical forms of *M. ozzardi* from the Peruvian villages in which Marcos *et al*.^8^ collected their samples have been previously reported^36^ and thus we felt it important to try to eliminate the possibility that the observed differences in CO1 barcodes was on account of them deriving from distinct strains or species. We did this by obtaining 12 S (mitochondrial DNA sequences) and 5 S nuclear ribosomal DNA sequences from our Brazilian *M. ozzardi* samples and using them in phylogenetic analysis with the 12 S and 5 S sequences that Marcos *et al*.^8^ obtained from the same Peruvian *M. ozzardi* samples which they recovered what we are contending here maybe CO1 Numts. Figure 5 shows a phylogenetic tree constructed using the mitochondrial 12 S sequences from a similar set of filarial parasite species samples as was used to prepare the CO1 tree. In contrast to the tree constructed with the CO1 gene, the 12 S mitochondrial sequence tree does not separate the Peruvian, Haitian and Brazilian *M. ozzardi* sequences into discrete clusters and displays very short branch lengths (genetic distances) between the *M. ozzardi* 12 S sequences. Across the 389 nucleotide positions used to construct the tree in Fig. 4, none of the *M. ozzardi* derived sequences (from Haiti, Brazil and Peru) share less than 97% identity (see supplementary information Fig. 4). Across the same 389 nucleotide sequence region *Wuchereria bancrofti* shares 92–95% identity with *Brugia* genus 12 S sequences. Consistent with the conclusions of Marcos *et al*.^8^, thus our mitochondrial 12 S mitochondrial DNA phylogenetic analysis suggest that none of the Peruvian *M. ozzardi* from their analysis are from an a-typical form of *M. ozzardi* and that *M. ozzardi* from across the Amazon region and the Caribbean are all very closely related. The nuclear ribosomal 5 S gene tree shown in Fig. 6 also strongly supports these conclusions. All of the *M. ozzardi* 5 S sequences (from Brazil, Peru and Bolivia) used to construct the tree in Fig. 5 cluster together tightly in a single bootstrap-supported group, in which no two sequences share less than 96% identity (see supplementary information Fig. 5).

## Discussion

### The complete mitogenome of *M. ozzardi* is a potentially valuable new resource for the filarial parasite research

Thus far almost all the molecular tools that have been developed to assist with filarial parasite research have been based on the PCR amplification and sequencing of a few select mitochondrial and ribosomal DNA taxonomic marker sequences^2,3^. In the field of mansonellosis research tools of this type have been used to: confirm the existence of *M. perstans* in Latin America^11^; invalidate the legitimacy of reports of a-typical forms of *M. ozzardi* parasites^8,30,37^ and to reveal the existence of a previously unrecognised subspecies of *M. perstans*, e.g. *M. perstans* “deux”^38^. Existing tools have not, however, yet provided much help in understanding of the phylogeography or population genetics of filarial parasites because the sequences that have been historically targeted have uncovered so little within species diversity^2,3^. With many intergenic and poorly conserved sequence regions, mitogenome sequences have the potential to be a resource from which to develop new molecular tools to study the phylogeography and population genetics of filarial parasites^34,39^. After determining and then aligning the whole mitogenome sequence of *D. repens* to all previously published Onchocercidae mitogenomes, Yilmaz *et al*.^27^ identified hypervariable regions and then used the mitogenome sequence of *D. repens* to design PCR primers to amplify these regions for a population genetics study. The *M. ozzardi* mitogenome reported here could, thus, be used to develop similar tools to the study the phylogeography and population genetics of *M. ozzardi* in much the same way. Furthermore, our finding here that *M. ozzardi* shares near perfect gene content, gene order and gene orientation with all previously published Onchocercidae mitogenomes suggests that generic pan-*Mansonella* genus or even pan-Onchocercidae family population genetics tools with the ability to amplify variable regions from a range of species could be designed from filarial parasite mitogenome alignments. Our discovery of multiple *M. ozzardi* pseudogene sequences (explained in more detail below), however, also shows that any such tools must be designed with extreme care.

### Whole mitogenome phylogenetic analysis raises questions about the placement of the *Mansonella* genus within ONC5

Overall, our phylogenetic analysis agrees well with the previous MLST analysis of Lefoulon *et al*.^35^, which found strong support for the monophyly of Onchocercidae and reported the existence of five well supported clades within it (ONC1 to ONC5). Our analysis, like the analysis of Yilmaz *et al*.^34^, provides strong support for at least the ONC4 and ONC5 clades that Lefoulon *et al*.^35^ reported and provides no indication that the other three clades that Lefoulon *et al*.^35^ reported are not equally valid. There are, however, a few minor within clade incongruences between our phylogeny and that of Lefoulon *et al*.^35^. Most notably, from a public health perspective, our mitogenome phylogeny (see Fig. 2) provides weak bootstrap support for the existence of monophyletic group that includes *M. ozzardi* and the filarial parasites that cause lymphatic filariasis (i.e those from the *Wuchereria* and *Brugia* genera)^35^, but which excludes *L. loa*. Our analysis thus suggests that *M. ozzardi* is more closely related to the lymphatic filariasis causing parasites than *L. loa* is, which contrasts with the MLST analysis of Lefoulon *et al*.^35^ that suggested they are equally distant (by very robustly clustering the *L. loa* parasite and *Mansonella* parasites together in a monophyletic group that excluded the lymphatic filariasis causing parasites). As our analysis was inferred entirely from mitochondrial DNA sequences, whereas the analysis of Lefoulon *et al*.^35^ was based on a mixture of mitochondrial and nuclear DNA sequences, their analysis can be considered less susceptible to the corrupting effects of the parasite´s maternally inherited endosymbiont, *Wolbachia*. It should, however, be noted that there are other important differences between our analysis and the analysis of Lefoulon *et al*.^35^ which could also be contributing to incongruences. Lefoulon *et al*.^35^ used nucleotide sequence alignments, which when used to infer phylogenies of distant taxa, can be more susceptible to effects of homoplasy than amino acid alignments. Yilmaz *et al*.^34^ who used some of the same gene sequences that Lefoulon *et al*.^35^ used, found significant support for saturation of nucleotide variation at the third codon position of the protein coding mitochondrial genes they analysed and for this reason chose (like us) to construct their trees with amino acid sequence alignments^34^. As *L. loa* parasites can compromise the reliability of the ICT cards, which are a popular immunodiagnostic tool that has been used extensively for the design and implementation of Lymphatic filariasis control and elimination programmes, the relationship these parasites have with *Mansonella* parasites (which are among the most common causes of human parasitaemias) is arguably thus a relationship that merits further investigation^40–42^.

### The identification of cryptic CO1 pseudogenes among GenBank sequence deposits

Using ribosomal DNA ITS-1 sequence it has been shown that *M. ozzardi* from Argentina, Roraima state (in the extreme north of Brazil), Acre and various parts of the Brazilian state of Amazonas are also very closely related^23,37,43^. Using the ribosomal 5 S gene here we have shown that *M. ozzardi* parasites from Peru, Bolivia and the Brazilian state of Amazonas are all very closely related too (see Fig. 4). In Fig. 2 our analysis of the 12 S mitochondrial gene shows that *M. ozzardi* from the Caribbean are also very closely related to *M. ozzardi* parasites from the Amazon region suggesting that *M. ozzardi* from the Caribbean are not a distinct species as it was once proposed they might be^4,5^. Our analysis of the Brazilian and the Haitian CO1, which suggested they are almost identical, adds further weight to this notion and thus our data can be taken collectively to strongly support the notion that there is just one species of *M. ozzardi* in the New World.

Given that both the mitochondrial 12 S gene and the nuclear 5 S genes of the Peruvian *M. ozzardi* parasites so strongly suggest they are the same parasite species, the distinct clustering of the Peruvian CO1 sequences in Fig. 2 must be viewed as anomalous. Reports of a single species, with very little nuclear or morphological variation, having multiple distinct CO1 barcodes have been made^44,45^. These reports are usually attributed to the effects of maternally inherited endosymbionts (like *Wolbachia*) which cause indirect selective sweeps on mitochondrial diversity^45^. Such phenomena, however, are expected to affect the whole mitogenome sequence and thus would not be expected to create *M. ozzardi* populations with distinct CO1 sequences, but near identical 12 S sequences and thus cannot easily explain our observed results^45^. More than 4 million morphologically identified species have had their CO1 barcodes deposited in public databases, but we are unaware of a single report of a species that has two or more distinct CO1 haplotypes (i.e CO1 sequences that vary by >15%) and which also has < 1% diversity across its mitochondrial 12 S gene sequence^46^. We believe that this and the fact that Peruvian *M. ozzardi* CO1 sequence deposits showed all the hall marks of mtDNA pseudogene (including possible frame-shift mutations) and half of the pseudogene contigs over 200 nucleotides (see table one) that we identified in our shot-gun sequence library screen were of CO1-origin, means that the most parsimonious explanation of the phylogenetic grouping of Peruvian CO1 sequences shown in Fig. 3 is that they are of non-coding mtDNA pseudogene origin. As, however, these Peruvian CO1 sequences appear to encode open reading frame sequences, it is not presently possible for us to entirely exclude the possibility that these Peruvian CO1 sequences encode functional proteins and thus that they represent a distinct variant CO1 allele rather than a cryptic CO1 pseudogene.

Most CO1 pseudogene sequences, which occur quite commonly across a range of species^17–20^ are of Numt origin. Because Numts that do not encode stop codons can be easily miss-identified as coding sequences^19^, we believe our results highlight the need for the careful screening of mitochondrial sequence for cryptic pseudogenes prior to their depositing in public databases. We believe that they also highlight the risk that mtDNA pseudogenes, like Numts, pose to the popular single-gene mtDNA diagnostics system of CO1 barcoding^13^. In our study half of the mtDNA pseudogenes we identified from our contig library were of CO1 sequence origin. By contrast we did not: detect any mitochondrial 12 S Numt sequences in our contig library; encounter difficulties Sanger sequencing this gene or indeed encounter sequence variants in GenBank. This suggests that the 12 S gene is a more reliable target for *M. ozzardi* diagnostics and systematics than the CO1 gene is. In Lamberton *et al*.^46^ they amplified three mitochondrial DNA targets (ND5 12 S and 16 S) to detect the presence of *O. volvulus* parasites in their insect vectors. Because of difficulties in sequencing the ND5 gene, which they did not encounter with the 12 S and 16 S genes, they stopped targeting the ND5 gene sequence. Such a scenario could be explained by the occurrence of Numts in the *O. volvulus* and it may thus be that there is something special about filarial parasite mtDNA 12 S genes that makes them less susceptible to forming the kind of Numts that affect their utility as population genetics tools. Given the potential value of mitogenomes and mtDNA-based tools to the field of filarial parasite research, we believe further investigations into filarial Numt diversity and prevalence are warranted. We believe also that it is worth noting that Numt sequences themselves can be used for the design of population genetics studies^47,48^. From our work presented here, for example, it appears that the Peruvian CO1 sequences of Marcos *et al*.^8^, may not just be cryptic Numts but might also be Numts which occur only in a geographically restricted range of parasites and thus could be a potentially useful resource for future phylogeographic and population genetics studies of *M. ozzardi*.

## Electronic supplementary material

Supplementary Information S1-5
